# Adverse drug effects observed with vildagliptin versus pioglitazone or rosiglitazone in the treatment of patients with type 2 diabetes mellitus: a systematic review and meta-analysis of randomized controlled trials

**DOI:** 10.1186/s40360-017-0175-0

**Published:** 2017-10-23

**Authors:** Pravesh Kumar Bundhun, Girish Janoo, Abhishek Rishikesh Teeluck, Feng Huang

**Affiliations:** 1 Institute of Cardiovascular Diseases, the First Affiliated Hospital of Guangxi Medical University, Nanning, Guangxi 530021 People’s Republic of China; 2 Guangxi Medical University, Nanning, Guangxi 530027 People’s Republic of China; 3 Institute of Cardiovascular Diseases and Guangxi Key Laboratory Base of Precision Medicine in Cardio-cerebrovascular Diseases Control and Prevention, the First Affiliated Hospital of Guangxi Medical University, Nanning, Guangxi 530021 People’s Republic of China

**Keywords:** Vildagliptin, Pioglitazone, Type 2 diabetes mellitus, Adverse drug events, Peripheral edema, Weight gain

## Abstract

**Background:**

Vildagliptin and pioglitazone/rosiglitazone are emerging Oral Hypoglycemic Agents (OHAs) which are used to treat patients suffering from Type 2 Diabetes Mellitus (T2DM). In this analysis, we aimed to systematically compare the adverse drug events which were observed with the use of vildagliptin versus pioglitazone or rosiglitazone respectively.

**Methods:**

Online databases were searched for studies comparing vildagliptin with pioglitazone/rosiglitazone. Adverse drug events were considered as the clinical endpoints in this analysis. We calculated Odds Ratios (OR) with 95% Confidence Intervals (CIs) using the RevMan 5**.**3 software. All the authors had full access to the data which were used and approved the final version of the manuscript.

**Results:**

A total number of 2396 patients were analyzed (1486 and 910 patients were treated with vildagliptin and pioglitazone/rosiglitazone respectively). Vildagliptin and pioglitazone/rosiglitazone were both associated with similar overall adverse drug events (OR: 1.00, 95% CI: 0.81–1.24; *P* = 1.00). Headache (OR: 0.88, 95% CI: 0.60–1.27; *P* = 0.49) and upper respiratory tract infection (OR: 0.95, 95% CI: 0.71–1.27; *P* = 0.75) were similarly observed. However, dizziness was significantly lower with pioglitazone/rosiglitazone (OR: 0.63, 95% CI: 0.43–0.92; *P* = 0.02).

Back pain, diarrhea and nausea were insignificantly lower with pioglitazone/rosiglitazone (OR: 0.81, 95% CI: 0.49–1.33; *P* = 0.40), (OR: 0.83, 95% CI: 0.48–1.44; *P* = 0.52) and (OR: 0.52, 95% CI: 0.25–1.05; *P* = 0.07) respectively, whereas peripheral edema and weight gain were insignificantly higher (OR: 1.21, 95% CI: 0.56–2.62; *P* = 0.63) and (OR: 2.29, 95% CI: 0.51–10.34; *P* = 0.28) respectively.

Nevertheless, when pioglitazone and rosiglitazone were separately compared with vildagliptin, peripheral edema and weight gain were significantly higher with rosiglitazone (OR: 2.36, 95% CI: 1.40–3.99; *P* = 0.001) and (OR: 5.20, 95% CI: 2.47–10.92; *P* = 0.0001) respectively.

**Conclusion:**

Both vildagliptin and pioglitazone/rosiglitazone are acceptable for the treatment of patients with T2DM on the basis that they are not significantly different in terms of overall adverse drug events. However, weight gain and peripheral edema would have to be re-assessed in further larger randomized controlled trials.

## Background

Vildagliptin, an inhibitor of dipeptidyl peptidase 4 (DPP-4), is a new Oral Hypoglycemic Agent (OHA) with a dual function. It is used to treat patients with Type 2 Diabetes Mellitus (T2DM) [1]. It induces the secretion of insulin in order to decrease blood sugar level (by inhibiting the inactivation of GLP-1 and GIP). It can also suppress glucagon release in the pancreas in order to prevent the release of glucose in blood [2]. Currently, this drug is approved for use especially in the European Union, Latin America and Asia. Vildagliptin, which is absorbed rapidly upon administration, is used alone or in combination with other OHAs.

Pioglitazone and rosiglitazone, of the thiazolidinedione class, are other OHAs which work by helping to restore the body’s proper response to insulin (by selectively stimulating peroxidase proliferator-activated receptor gamma [PPAR-ℽ and PPAR-α]), thereby lowering blood sugar [3, 4]. They have often been used in combination with metformin, sulfonylurea and insulin.

The main adverse drug events which have been reported with the use of vildagliptin include nausea, peripheral edema, weight gain, headache, dizziness, upper respiratory infection, back pain and diarrhea. Similar adverse drug events have also been observed with the use of pioglitazone and rosiglitazone.

With these emerging OHAs in the market, having a common aim to decrease the blood sugar level in patients with T2DM, it is high time to compare their adverse effects.

In this meta-analysis, we aimed to systematically compare the adverse drug events which were observed with the use of vildagliptin versus pioglitazone or rosiglitazone respectively in patients who were treated for T2DM.

## Methods

### Data sources and search strategy

Online databases including EMBASE, MEDLINE and the Cochrane library were searched for studies comparing vildagliptin with pioglitazone or rosiglitazone for the treatment of patients with T2DM by typing the words or phrases ‘vildagliptin and pioglitazone/rosiglitazone’. The abbreviation ‘T2DM’ was also included in this search process.

### Inclusion and exclusion criteria

Studies were included if:

- They were randomized controlled trials (RCTs).
- They were articles which were published in English language.
- They compared vildagliptin with either pioglitazone or rosiglitazone.
- They reported adverse drug events.
- They consisted of patients with T2DM.

Studies were excluded if:

- They were meta-analyses, observational studies, case studies or letter to editors.
- They did not compare vildagliptin with pioglitazone or rosiglitazone.
- They did not report adverse drug events (since the main focus was on adverse drug events).
- They were duplicated studies.

### Endpoints and follow ups

Endpoints which were assessed included:

-Any adverse drug event (any type of adverse drug event);

-Nausea;

-Upper respiratory infection including naso-pharyngitis;

-Weight gain;

-Headache;

-Dizziness;

-Peripheral edema;

-Back pain;

-Diarrhea.

The adverse drug outcomes which were reported in each study were summarized in Table 1.

**Table 1:** Adverse drug events which were reported

Studies	Adverse events reported	Follow up period
Bolli2008	Peripheral edema, headache, nasopharyngitis, back pain, dizziness, diarrhea	52 weeks
Garber2006	Any AE, peripheral edema, weight increased, headache, dizziness, nausea	24 weeks
Kim2010	Any AE, nasopharyngitis, dizziness, headache, upper respiratory infection, headache	24 weeks
Rosenstock2007	Any AE, nasopharyngitis, dizziness, headache, upper respiratory tract infection, peripheral edema, weight gain	24 weeks
Rosenstock2009	Any AE, Nasopharyngitis, dizziness, upper respiratory tract infection, arthralgia, back pain, headache, nausea, diarrhea, peripheral edema, weight increased	104 weeks

*Abbreviations*: *UTI* urinary tract infection, *SAEs* serious adverse events, *AE* adverse event

### Data extraction and review

Three authors (P.K.B, G.J and A.R.T) independently reviewed the data, analyzed the type of study and then assessed the eligibility and methodological quality of each selected trial. The authors’ names, year of publication, the total number of patients who were treated with vildagliptin and pioglitazone/rosiglitazone respectively, their baseline characteristics, and the reported adverse drug events were all systematically extracted accordingly. These data were further cross-checked by the fourth author (F.H). The bias risk was assessed in collaboration with Cochrane [5].

### Assessment of heterogeneity, reported bias and statistical analysis

The Preferred Reporting Items for Systematic Reviews and Meta-Analyses guideline was followed [6]. Heterogeneity across the trials was assessed first of all using the Cochrane Q-statistic test (*p* ≤0**·**05) and then by the I^2^-statistic test. An I^2^ value with low percentage represented a lower heterogeneity. A fixed (I^2^ < 50%) or a random (I^2^ > 50%) effects model was used accordingly during the subgroup analysis. Publication bias was visually estimated by assessing funnel plots. Odd Ratios (OR) and 95% Confidence Intervals (CIs) were calculated using the RevMan 5.3 software.

## Results

### Selection of studies

**Fig. 1:**
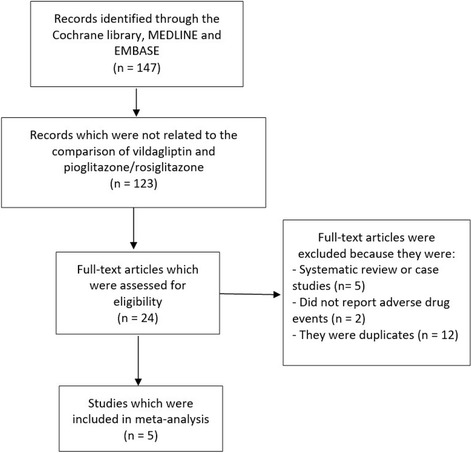
Flow diagram representing the study selection

One hundred and forty-seven (147) articles were searched from Online databases. One hundred and twenty-three (123) articles were eliminated on the basis of abstracts and titles. Twenty-four (24) full text articles were assessed for eligibility. Five articles were further eliminated since they were case studies or meta-analyses. Two studies did not report the corresponding adverse drug events and were therefore eliminated because our main focus was only on studies that reported adverse drug events. Twelve (12) articles were duplicated studies and they were therefore eliminated. Finally, five trials [7–11] were selected for this meta-analysis (Fig. 1).

### General features of the trials which were included in this analysis

A total number of 2396 patients were analyzed (1486 patients were treated with vildagliptin whereas 910 patients were treated with either pioglitazone or rosiglitazone) [Table 2]. All the studies which were included in this analysis were RCTs. Trial Rosenstock2007 consisted of the largest number of patients in comparison to the other trials. Three studies reported the use of pioglitazone whereas two other studies reported the use of rosiglitazone.

**Table 2:** General features of the studies which were included

Studies	Type of study	No of patients treated with vildagliptin (n)	No of patients treated by pioglitazone or rosiglitazone (n)	Drug used in experimental/control groups
Bolli2008 [7]	RCT	295	280	Pioglitazone/vildagliptin
Garber2006 [8]	RCT	304	158	Pioglitazone/vildagliptin
Kim2010 [9]	RCT	35	36	Pioglitazone/vildagliptin
Rosenstock2007 [10]	RCT	459	238	Rosiglitazone/vildagliptin
Rosenstock2009 [11]	RCT	393	198	Rosiglitazone/vildagliptin
Total no of patients (n)		1486	910	

*Abbreviations*: *RCT* randomized controlled trials

### Baseline features of the trials which were included

As shown in Table 3, mean age of the patients, the total number of male patients, and the percentage range of HbA1c were not significantly different between patients who were treated with vildagliptin versus pioglitazone or rosiglitazone respectively. Duration of T2DM varied from 1.98 years to 6.4 years.

**Table 3:** Baseline features of the patients

Studies	Mean age (y)	Males (%)	HbA1c (%)	Duration of DM (y)	Bias risk assessment
	Exp/cont	Exp/cont	Exp/cont	Exp/cont	
Bolli 2008	57.0/56.3	64.1/61.7	8.4/8.4	6.4/6.4	B
Garber 2006	54.8/54.0	50.7/49.9	8.7/8.65	4.8/4.75	B
Kim 2010	49.2/49.8	83.3/88.9	8.7/8.6	2.1/1.9	B
Rosenstock 2007	54.2/54.5	57.6/57.5	8.7/8.7	2.7/2.3	B
Rosenstock 2009	54.2/54.4	54.5/57.6	8.63/8.58	2.60/1.98	B

*Abbreviations*: *Exp* experimental group (pioglitazone or rosiglitazone), *Cont* control group (vildagliptin), *y* year, *DM* diabetes mellitus

Other OHAs which were used, and the dosages of vildagliptin versus pioglitazone/rosiglitazone have been represented in Table 4.

**Table 4:** Other oral anti-hypoglycemic agents and dosages of vildagliptin and pioglitazone/rosiglitazone which were reported

Trials	Other OHA which were used with vildagliptin	Other OHA which were used with pioglitazone/rosiglitazone	Dosage of vildagliptin	Dosage of pioglitazone/rosiglitazone
Bolli 2008	Metformin	Metformin	50 mg bd	30 mg bd
Garber 2006	Pioglitazone	–	50 or 100 mg qd	45 mg qd
Kim 2010	–	–	100 mg qd	30 mg qd
Rosenstock 2007	–	–	100 mg qd	8 mg od
Rosenstock 2009	–	–	50 mg bd	8 mg qd

*Abbreviations*: *OHA* oral hypoglycemic agents

### Comparing the adverse drug events

Results of this analysis have been tabulated (Table 5).

**Table 5:** Results of this analysis

Endpoints	OR with 95% CI	*P* value	I^2^ (%)
Any AE	1.00 [0.81–1.24]	1.00	0
Headache	0.88 [0.67–1.27]	0.49	0
Nasopharyngitis/URI	0.95 [0.71–1.27]	0.75	6
Back pain	0.81 [0.49–1.33]	0.40	0
Dizziness	0.63 [0.43–0.92]	0.02	0
Diarrhea	0.83 [0.48–1.44]	0.52	0
Nausea	0.52 [0.25–1.05]	0.07	0
Weight gain	2.29 [0.51–10.34]	0.28	80
Peripheral edema	1.21 [0.56–2.62]	0.63	76

Nasopharyngitis and upper respiratory tract infection were combined together during the analysis

*Abbreviations*: *URI* upper respiratory infection, *AE* adverse events, *OR* odds ratio, *CI* confidence interval

**Fig. 2:**
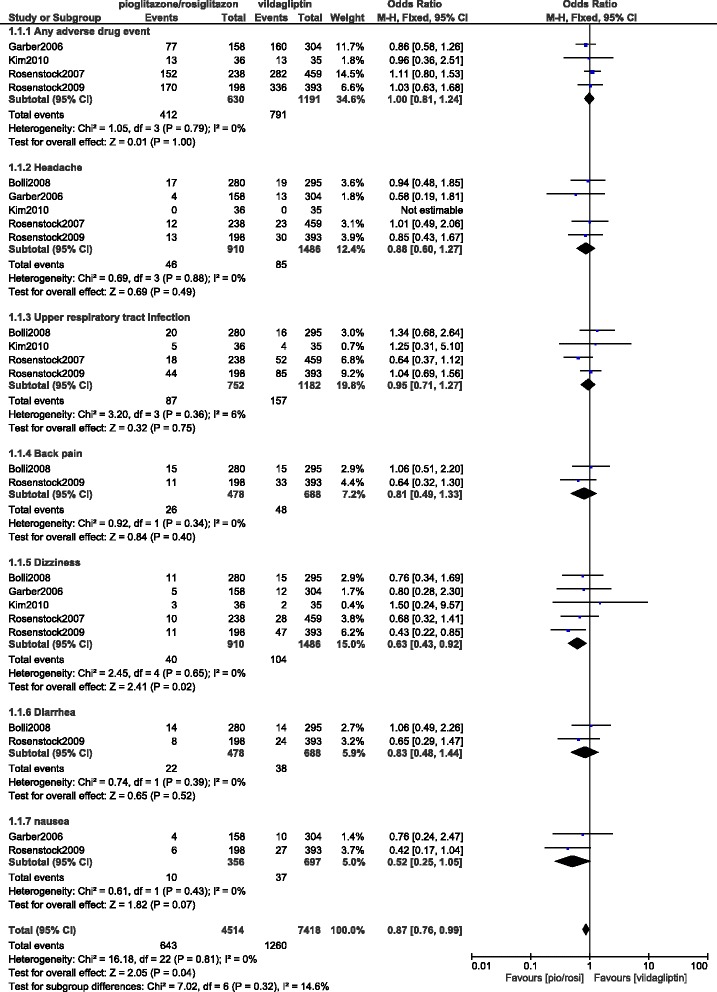
Adverse drug events reported between vildagliptin and pioglitazone/rosiglitazone (part 1)

Similar overall adverse drug events were observed with vildagliptin and pioglitazone or rosiglitazone (OR: 1.00, 95% CI: 0.81–1.24; *P* = 1.00). When the adverse drug events were further subdivided, a similar headache and upper respiratory tract infection were observed (OR: 0.88, 95% CI: 0.60–1.27; *P* = 0.49) and (OR: 0.95, 95% CI: 0.71–1.27; *P* = 0.75) respectively. Dizziness was significantly lower with pioglitazone/rosiglitazone (OR: 0.63, 95% CI: 0.43–0.92; *P* = 0.02). Even if back pain, diarrhea and nausea were lower in patients who were treated with pioglitazone/rosiglitazone (OR: 0.81, 95% CI: 0.49–1.33; *P* = 0.40), (OR: 0.83, 95% CI: 0.48–1.44; *P* = 0.52) and (OR: 0.52, 95% CI: 0.25–1.05; *P* = 0.07) respectively, the results were not statistically significant. Results for the adverse drug events which were reported with vildagliptin and pioglitazone/rosiglitazone have been represented in Fig. 2.

**Fig. 3:**
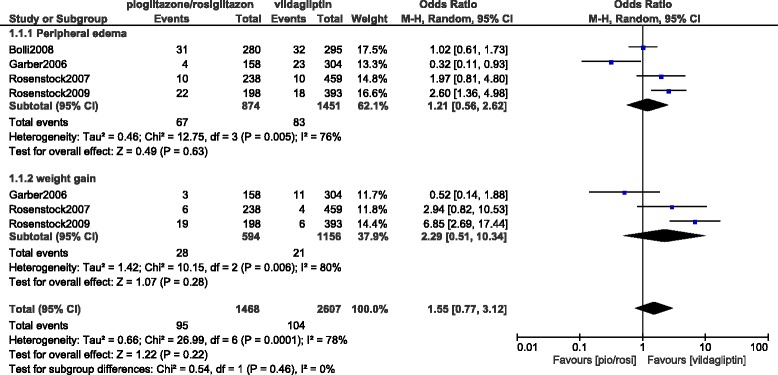
Adverse drug events reported between vildagliptin and pioglitazone/rosiglitazone (part 2)

Peripheral edema and weight gain were insignificantly lower with vildagliptin (OR: 1.21, 95% CI: 0.56–2.62; *P* = 0.63) and (OR: 2.29, 95% CI: 0.51–10.34; *P* = 0.28) respectively. Results analyzing these two outcomes have been represented in Fig. 3.

Another analysis was carried out comparing pioglitazone and rosiglitazone individually with vildagliptin.

**Fig. 4:**
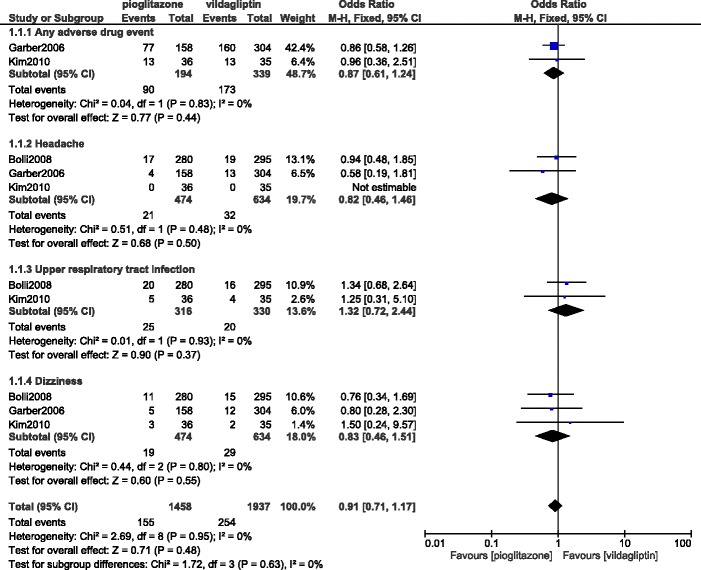
Adverse drug events reported between vildagliptin versus pioglitazone alone (part 1)

When pioglitazone was separately compared with vildagliptin, overall adverse drug events, headache, upper respiratory tract infections, and dizziness were not significantly different with (OR: 0.87, 95% CI: 0.61–1.24; *P* = 0.44), (OR: 0.82, 95% CI: 0.46–1.46; *P* = 0.50), (OR: 1.32, 95% CI: 0.72–2.44; *P* = 0.37), and (OR: 0.83, 95% CI: 0.46–1.51; *P* = 0.55) respectively. The adverse drug events which were reported between vildagliptin and pioglitazone alone have been represented in Fig. 4.

**Fig. 5:**
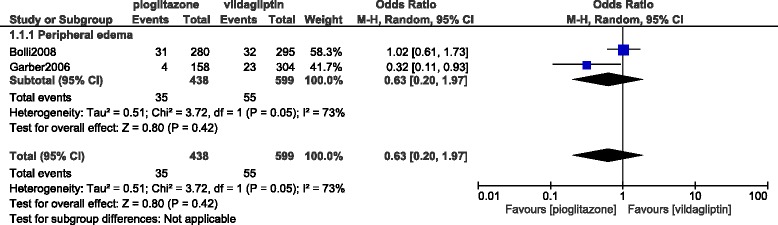
Adverse drug events reported between vildagliptin versus pioglitazone alone (part 2)

Peripheral edema was also not significantly different with (OR: 0.63, 95% CI: 0.20–1.97; *P* = 0.42) as shown in Fig. 5.

**Fig. 6:**
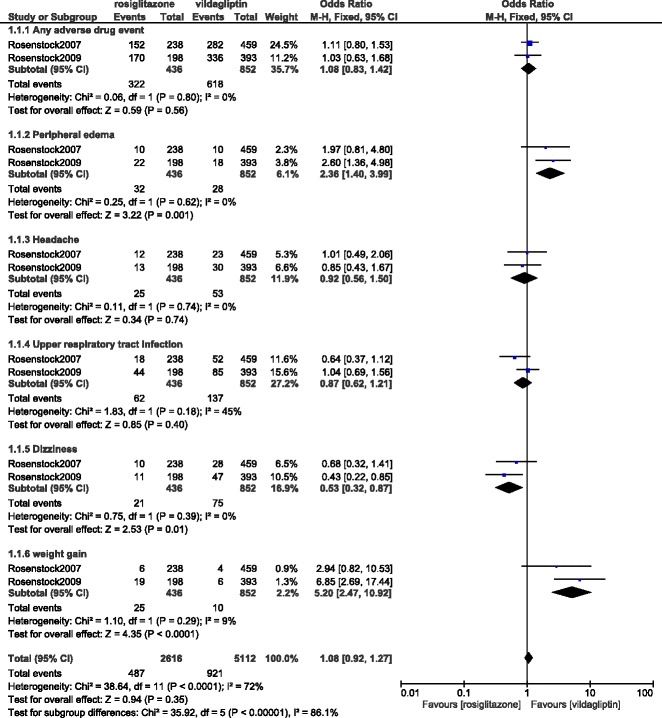
Adverse drug events reported between vildagliptin versus rosiglitazone alone

However, when rosiglitazone was separately compared with vildagliptin, even if ‘any adverse events’ was not significantly different (OR: 1.08, 95% CI: 0.83–1.42; *P* = 0.56), peripheral edema and weight gain were significantly higher with rosiglitazone (OR: 2.36, 95% CI: 1.40–3.99; *P* = 0.001) and (OR: 5.20, 95% CI: 2.47–10.92; *P* = 0.0001) respectively. However, dizziness was significantly higher with vildagliptin (OR: 0.53, 95% CI: 0.32–0.87; *P* = 0.01) whereas upper respiratory tract infection and headache were not significantly different. The adverse drug events which were reported between vildagliptin and rosiglitazone alone have been represented in Fig. 6.

**Fig. 7:**
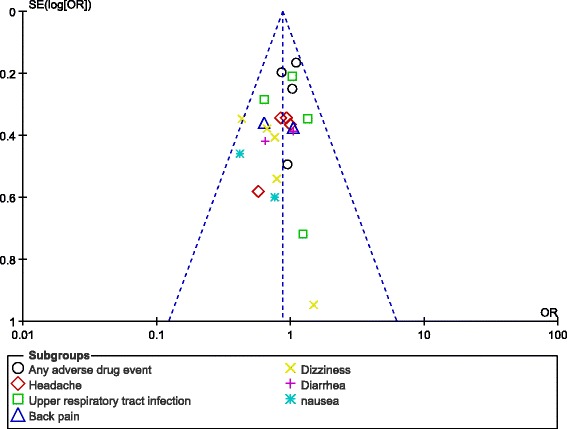
Funnel plot representing publication bias

As previously mentioned, publication bias was assessed by observing funnel plots. Bias is likely to show asymmetry in such plots. Other methods such as Begg-Mazumdar, Egger and Harbord-Egger tests were not carried out since they would not show better results compared to the funnel plots due to a very small volume of studies which was used. After visually assessing the funnel plot, a very low publication bias was observed among the studies which were used to assess the adverse drug events associated with vildagliptin versus pioglitazone/rosiglitazone (Fig. 7).

## Discussion

Vildagliptin and pioglitazone/rosiglitazone did not show any significant difference in overall adverse drug events during a follow up period ranging from 24 to 104 weeks. However, when pioglitazone and rosiglitazone were separately compared with vildagliptin, rosiglitazone was associated with significantly higher peripheral edema and weight gain.

Similar to this current analysis, the trial comparing vildagliptin and pioglitazone in patients with impaired glucose tolerance following kidney transplantation demonstrated that both vildagliptin and pioglitazone were beneficial in such types of patients [12]. Mild adverse drug events were equally reported between these two drug types.

Rosenstock et al. showed a higher rate of peripheral edema and weight gain which were associated with rosiglitazone compared to vildagliptin in these patients with T2DM. Previous studies also showed edema to be among the major side effects of rosiglitazone and weight gain was also associated with this medication due to water retention from edema [13].

In addition, other studies have shown peripheral edema to manifest most often when pioglitazone/rosiglitazone was combined with other OHAs such as metformin, sulfonylurea or insulin [14]. Nevertheless, further studies with a large number of patients will be able to completely solve this issue. In addition, dosage of drug is another factor which should be considered in future studies, similar to the recent study which was published by Bundhun’s et al. which showed adverse drug events that were reported in patients who were treated with 100 mg versus 300 mg canagliflozin [15].

This is the first analysis to systematically compare the adverse drug events which were observed with vildagliptin versus pioglitazone or rosiglitazone in patients who were treated for T2DM.

### Limitations

Similar to other studies, this study also has limitations. First of all, due to the limited number of patients which were analyzed, this research might not provide a robust result. In addition, several adverse drug events were not analyzed since they were not repeatedly reported in other studies. Moreover, the follow up periods have been neglected in this analysis. This could also have had an effect on the overall results. Also, in a few studies, patients were also being treated with metformin which could have influenced the results in one way or the other. Furthermore, the dosage of drug was not similar in all of the studies and this might also be considered as a limitation.

## Conclusion

Both vildagliptin and pioglitazone/rosiglitazone are acceptable for the treatment of patients with T2DM on the basis that they are not significantly different in terms of overall adverse drug events. However, weight gain and peripheral edema would have to be re-assessed in further larger randomized controlled trials.

## Abbreviations

OHAs: oral hypoglycemic agents
T2DM: type 2 diabetes mellitus
